# A Gastrointestinal Stromal Tumor (GIST) and a Pseudocyst of the Pancreas: A Peculiar Case of Both Co-existing in the Same Patient

**DOI:** 10.7759/cureus.61642

**Published:** 2024-06-04

**Authors:** Manwar S Ali, Ankur Cheleng, Pravanjan Behera, Manash R Sahoo

**Affiliations:** 1 Surgery, All India Institute of Medical Sciences, Bhubaneswar, Bhubaneswar, IND; 2 General Surgery, All India Institute of Medical Sciences, Bhubaneswar, Bhubaneswar, IND

**Keywords:** cyst, stomach, mesenchymal tumors, pancreatic pseudocyst, gastrointestinal stromal tumor (gist)

## Abstract

Gastrointestinal stromal tumors (GISTs) are the most common mesenchymal neoplasms of the gastrointestinal (GI) tract, typically originating from the interstitial cells of Cajal. The clinical presentations are variable according to their size and shape but rarely present as a palpable abdominal mass.

Pancreatic pseudocysts are common complications of chronic pancreatitis characterized by fluid collections surrounded by a non-epithelialized wall of fibrous and granulation tissue. Patients may present with non-specific symptoms like abdominal pain, nausea, and vomiting and they generally have a history of acute pancreatitis. Small pseudocysts often resolve spontaneously, but larger ones often become symptomatic and may lead to complications.

It is rare to find both a GIST of the stomach and a pseudocyst of the pancreas in the same patient. We present a unique case of a giant GIST and a pancreatic pseudocyst in a 72-year-old male who was experiencing abdominal pain and distension. Imaging revealed a massive lesion originating from the posterior gastric wall, which resembled a pseudocyst, along with a distinct cystic lesion adjacent to the pancreatic body. During surgical exploration, a complex interplay of both pathologies was discovered, requiring a comprehensive resection approach. The successful outcome highlights the importance of careful evaluation and personalized management in such rare cases.

## Introduction

Gastrointestinal stromal tumors (GISTs), previously classified as leiomyomas, leiomyosarcomas, or leiomyoblastomas, constitute about 85% of all mesenchymal neoplasms affecting the gastrointestinal (GI) tract [1,2].

After the discovery of gain-of-function mutations in the c-KIT proto-oncogene by Hirota et al. in 1998, these tumors were identified as distinct histological subtypes of mesenchymal tumors [3,4].

They arise from KIT(CD117) (CD117)-positive interstitial cells of Cajal, the pacemaker cells of the GI tract, most commonly from the stomach, followed by the small intestine [5]. They are typically solid tumors without cystic changes [6]. The most frequent symptoms are anemia, weight loss, GI bleeding, and abdominal pain.

Additionally, they might have a presentation of an acute abdomen with obstruction, perforation, rupture, and peritonitis. Most GISTs are asymptomatic until they reach a significant size [5]. They are best identified by computed tomography (CT) and stain positive for CD117, CD34, and DOG1 [7-9].

Pseudocysts of the pancreas belong to the group of cystic lesions of the pancreas, which are generally sequelae of acute or chronic pancreatitis [10,11]. The incidence of pseudocysts has increased due to the widespread availability of diagnostic imaging techniques [10]. The fluid-filled collection, surrounded by a non-epithelialized wall of fibrous and granulation tissue, is formed when there is damage to the pancreatic ducts, usually from alcohol or biliary stones [10,11].

The advent of new endoscopic techniques of drainage has made the management of pseudocysts different and varies from patient to patient. The main indications of any type of drainage procedure are persistent patient symptoms and complications like hemorrhage, infection, and outlet or biliary obstruction. However, the surgical method of drainage remains the treatment of choice [12].

The existence of both of these conditions has not yet been seen or documented in the literature. Here we present a rare case of an extremely large GIST presenting as a palpable abdominal mass with fluid thrill, with predominantly cystic changes thought to be mimicking a large pseudocyst of the pancreas but rather existing simultaneously, which was cured with complete surgical resection.

## Case presentation

A 72-year-old male, a farmer by occupation, presented to the Surgery OPD with complaints of pain in the abdomen and abdominal distension for the past month. The pain was insidious in onset, diffuse, and vague, with no radiation or relation to food intake. The abdominal distension was progressive and associated with melaena. The Patient had no history of smoking or alcohol intake and had no family history of malignancies. There was no history of significant weight loss, vomiting, hematemesis, or jaundice. There is no past medical or surgical history.

**Figure 1:**
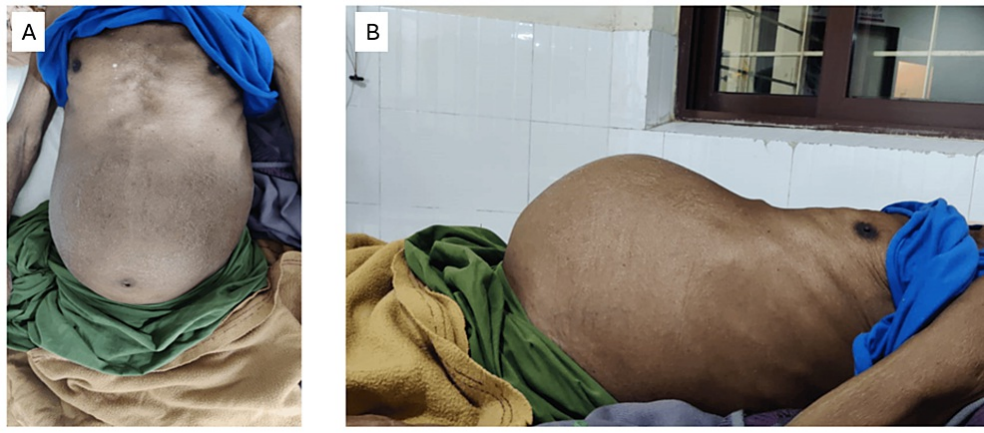
Pre-operative images of the abdomen (A) Front view and (B) lateral view.

On examination, the abdomen was distended with a visible fullness in the central and lower quadrant, with the umbilicus everted (Figure 1). On palpation, there was a 20 x 30 cm mass involving the umbilical region, epigastrium, hypogastrium, right and left lumbar, and iliac fossa. The mass had a cystic consistency with a smooth surface and well-defined margins, was not moving with respiration, and had a fluid thrill over the surface. There was no supraclavicular lymphadenopathy. The bowel sounds were normal. The digital rectal examination was within normal limits.

Ultrasonography of the whole abdomen and pelvis showed a pseudocyst of the pancreas. Contrast-enhanced computed tomography abdomen revealed an extensive heterogeneously enhancing lesion of size 20 x 22 x 28 cm, likely arising from the posterior wall of the stomach (likely GIST). Another well-defined peripherally enhancing cystic lesion measuring 20 x 20 x 22 cm was in contact with the above-mentioned lesion (likely cystic component/ pseudocyst of the pancreas) arising from the body of the pancreas. The lesion was causing a mass effect over the aorta, inferior vena cava (IVC), and left kidney. Multiple enhanced peripancreatic lymph nodes were noted.

Upper GI endoscopy showed a gastric bulge (likely extrinsic compression) of size 5 x 5 cm seen along the junction of the posterior wall and greater curvature. The mucosa overlying the bulge was edematous. Dilated gastric veins were present.

**Figure 2:**
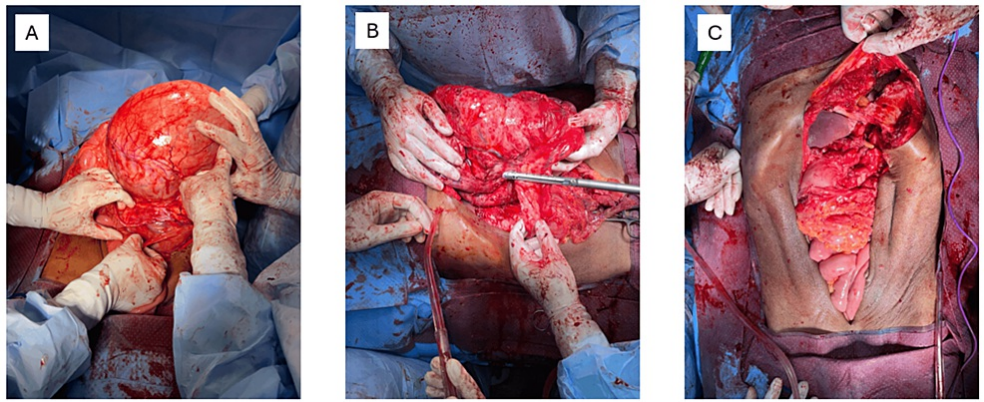
Intra-operative images. (A) Intra-operative mass arising from the posterior wall of the stomach. (B) Distal pancreatectomy done for the pseudocyst arising from the body and tail of the pancreas. (C) After resection of the intra-operative mass.

The patient was managed with an exploratory laparotomy via a thoraco-abdominal incision. Intra-operatively, a mass of size 20 x 20 x 20 cm was seen arising from the posterior wall of the stomach (Figure 2A), which adhered to the body and tail of the pancreas, along with the splenic vessels (Figure 2B). There was another cystic mass of size 15 x 15 x 18 cm communicating with the solid mass lesion. The cystic mass was aspirated and decompressed, which contained turbid brown fluid, after which the entire mass was resected. The wall of the cystic mass was sent for a frozen section, which was revealed to be benign and was preceded by a resection of the mass. The left thoracic cavity was entered into the process, which was managed by the repair of the diaphragm and intercostal drainage (ICD) placement (Figure 2C). 

The final procedure done was the resection of the gastric GIST with distal pancreatectomy along with splenectomy and resection of a segment of the transverse colon with repair of the posterior gastric wall sleeve and a colo-colic anastomosis. The total operative time was four hours and intra-operative blood loss was around 500 ml. The closure of the thoracoabdominal incision was done and an abdominal drain and an ICD were placed. The patient received two packed red blood cells post-operatively.

**Figure 3:**
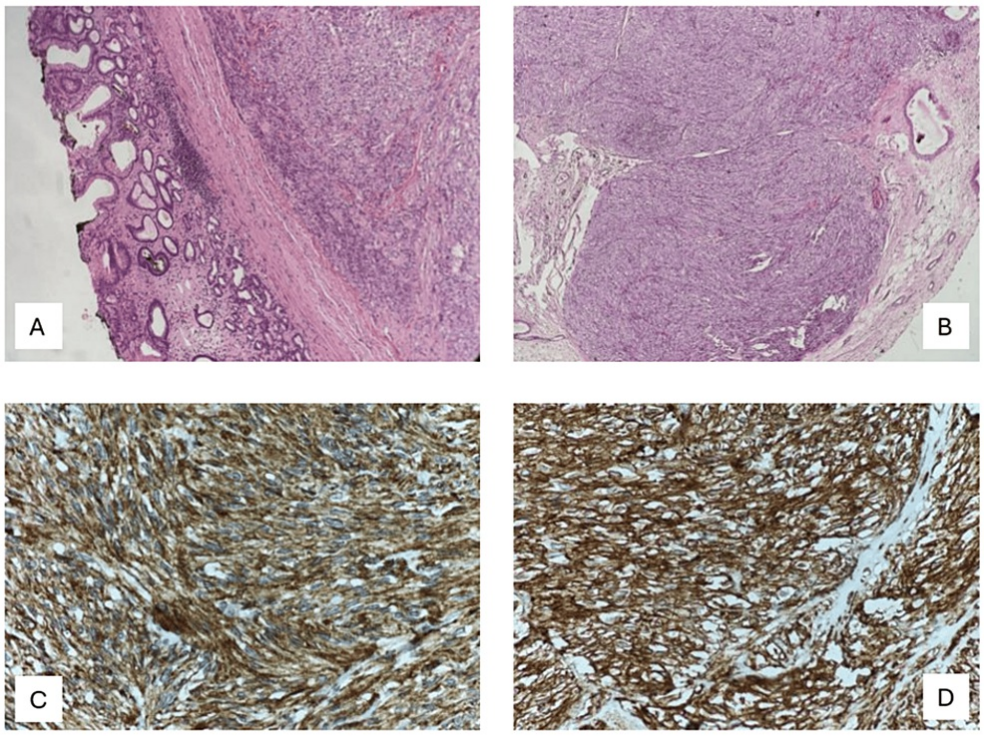
GIST arising from the posterior wall of the stomach. (A) Section from the gastric mass showing normal compressed gastric mucosa at one end and the tumor arising in the submucosa and muscularis propria. (B) The tumor is composed of cells arranged in nodules separated by fibrous stroma. (C) The cells are immunopositive for cKIT. (D) The tumor cells are immunopositive for DOG1. GIST: Gastrointestinal stromal tumor

**Figure 4:**
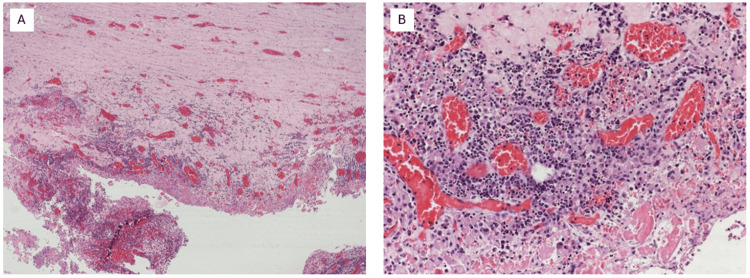
Pseudocyst arising from the body and tail of the pancreas. (A) A section of the cyst shows a fibro-collagenous wall. (B) The cyst does not have a lining epithelium. Its lumen shows inflammatory granulation tissue, plasma cells, and histiocytes.

The histopathology of the case showed both the GIST and pseudocyst of pancreas components which was an unusual finding. The GIST was seen arising from the submucosa and muscularis propria of the stomach (Figure 3). The wall of the cystic tumor arising from the pancreas did not have a lining epithelium (Figure 4).

The patient had an uneventful recovery after surgery and was discharged on the tenth post-operative day. Post-operatively, the abdominal wound was healthy and the patient was started on adjuvant imatinib therapy.

## Discussion

GISTs comprise 0.1 to 3% of all gastrointestinal tumors and are the most common type of mesenchymal tumor [5]. GISTs can present a wide range of non-specific clinical symptoms, including bleeding, intestinal obstruction, abdominal pain, palpable masses, and perforations, and may even be asymptomatic. As in this case, the diagnosis of GISTs in the stomach is often unclear pre-operatively.

Hepatic or pancreatic lesions can be misdiagnosed as GISTs of the stomach with cystic alterations, as they are uncommon [6]. GISTs that are more than 10 cm in size are invasive and have the potential to become malignant, and frequently spread to distant organs. Rarely do GIST metastases form in lymph nodes; instead, they typically occur in the liver and abdominal cavity [6].

The incidence of pseudocysts of the pancreas in acute pancreatitis ranges from 5% to 16%, whereas in chronic pancreatitis, the number ranges from 20% to 40%. The highest incidences are seen in alcoholic patients [12].

Chronic pseudocysts for more than eight weeks generally do not resolve spontaneously, and as the size increases (greater than 5 cm), the chance of complications increases too. Surgery, being the treatment modality of choice, has high success rates with low morbidity and mortality [10].

They have never been seen simultaneously in the same patient, with no previous case reports or literature proving their co-existence. The possible etiology of the co-existence of both in this patient might be the larger GIST compressing the body and tail of the pancreas, leading to the invasion and destruction of the ductal system and subsequent development of the pseudocyst. The patient had no prior history of alcoholism and biliary colic or radiological evidence of stones in the gallbladder or pancreas. 

In our case, the tumor was closely related to other organs like the spleen, left kidney, and IVC. It was indistinct from the pseudocyst arising from the body and the tail of the pancreas and was previously thought to be the same lesion. Although our patient was subjected to stomach endoscopy, CT scan, and abdominal ultrasound, these diagnostic techniques were unable to provide a conclusive diagnosis.

## Conclusions

To our knowledge, this is the first case report of a GIST and a pseudocyst of the pancreas existing in the same patient, and no other cases have been documented in the literature regarding the same. The possible reason for the co-existence of both these conditions is still not clear. One of the reasons might be the invasion and destruction of the ductal system of the body of the pancreas by the GIST over time. This eventually might have led to the formation of the pseudocyst from the body and tail of the pancreas. As the patient did not have any history of alcoholism or previous bouts of abdominal pain suggestive of biliary colic or pancreatitis, this seems to be an appropriate explanation for the co-existence. In any case of GIST and pseudocyst of the pancreas, the tumor size is the most important prognostic factor. Resection of both tumors, followed by adjuvant therapy with imatinib, was the preferred option for our patient. Follow-up for recurrence of the GIST of the stomach is the primary concern in our case.
